# Epidemiology of tuberculosis in Sabah, Malaysia, 2012–2018

**DOI:** 10.1186/s40249-020-00739-7

**Published:** 2020-08-26

**Authors:** Michelle May D. Goroh, Giri Shan Rajahram, Richard Avoi, Christel H. A. Van Den Boogaard, Timothy William, Anna P. Ralph, Christopher Lowbridge

**Affiliations:** 1 TB/Leprosy Control Unit, Sabah State Health Department, Kota Kinabalu, Malaysia; 2 Faculty of Medicine and Health Sciences, Universiti Malaysia Sabah, Kota Kinabalu, Malaysia; 3 Queen Elizabeth Hospital, Kota Kinabalu, Malaysia; 4 Menzies School of Health Research, Charles Darwin University, Darwin, Australia; 5 Infectious Diseases Society of Kota Kinabalu, Kota Kinabalu, Malaysia; 6 Gleneagles Hospital, Kota Kinabalu, Malaysia; 7 Division of Medicine, Royal Darwin Hospital, Darwin, Australia

**Keywords:** Tuberculosis, Epidemiology, Surveillance, Sabah, Malaysia

## Abstract

**Background:**

Tuberculosis (TB) is of high public health importance in Malaysia. Sabah State, located on the island of Borneo, has previously reported a particularly high burden of disease and faces unique contextual challenges compared with peninsular Malaysia. The aim of this study is to describe the epidemiology of TB in Sabah to identify risk groups and hotspots of TB transmission.

**Methods:**

We conducted a retrospective review of TB cases notified in Sabah, Malaysia, between 2012 and 2018. Using data from the state’s ‘myTB’ notification database, we calculated the case notification rate and described trends in the epidemiology, diagnostic practices and treatment outcomes of TB in Sabah within this period. The Chi-squared test was used for determining the difference between two proportions.

**Results:**

Between 2012 and 2018 there were 33 193 cases of TB reported in Sabah (128 cases per 100 000 population). We identified several geographic hotspots, including districts with > 200 cases per 100 000 population per year. TB rates increased with age and were highest in older males. Children < 15 years accounted for only 4.6% of cases. Moderate or advanced disease on chest X-ray and sputum smear positivity was high (58 and 81% of cases respectively), suggesting frequent late diagnosis. Multi-drug resistant (MDR) TB prevalence was low (0.3% of TB cases), however, rapid diagnostic test coverage was low (1.2%) and only 18% of all cases had a positive culture result. Treatment success was 83% (range: 81–85%) in those with drug-sensitive TB and 36% (range: 25–45%) in cases of MDR-TB.

**Conclusion:**

Between 2012 and 2018, TB notifications in Sabah State equated to 20% of Malaysia’s total TB notifications, despite Sabah representing only 10% of Malaysia’s population. We found hotspots of TB in urbanised population hubs and points of migration, as well as evidence of late presentation and diagnosis. Ensuring universal health coverage and expansion of GeneXpert® coverage is recommended to reduce barriers to care and early diagnosis and treatment for TB.

## Background

Tuberculosis (TB) is a disease of public health importance in Malaysia, with 25 173 cases of the disease recorded nationally in 2018, and an estimated incidence rate of 92 cases per 100 000 population [1]. Malaysia is classified by the World Bank as an upper-middle-income economy [2]. This can mask the reality of deep poverty affecting many residents of the eastern states of Malaysian Borneo. In the present era of the severe acute respiratory syndrome coronavirus 2 (SARS-CoV-2) pandemic, there has been strong recognition of the need for investment in early detection and prevention of transmissible respiratory disease, rather than reactive disease treatment [3]. These lessons, especially the proven effectiveness of case detection, case isolation and contract tracing, provide much-needed inspiration to re-orient healthcare services in high TB burden settings towards prevention. Examining regional TB epidemiology provides the best mechanism for assessing the effectiveness of existing prevention and management strategies.

While Malaysia is successfully reducing TB related mortality [1, 4], TB incidence is not decreasing in line with global End TB Strategy milestones [5]. Sabah State, located in Malaysian Borneo, has historically had a higher burden of TB. While Sabah State accounts for only 10% of the country’s total population, it has been reported to account for 20–30% of all Malaysia’s TB cases [6, 7]. While TB rates in other parts of the country have fallen over the past decade, sustained high TB case notification rates of 144 to 217 cases per 100 000 population have been observed [8]. Previous studies have indicated that the TB epidemic in Sabah is chiefly related to delayed health seeking and limited access to TB care, rather than factors such as HIV or drug resistance which may drive TB epidemics elsewhere [9]. Fortunately rates of HIV co-infection and drug-resistance in Sabah have been reported to be relatively low [9, 10]. However, presentations with pulmonary TB are often late, with extensive cavitary disease and profound weight-loss frequently evident at diagnosis [9]. Delayed diagnosis and advanced disease are likely to result in poorer patient outcomes and increased risk of transmission prior to starting treatment.

The World Health Organization’s End TB Strategy sets ambitious targets to which countries must strive in order to end the global TB epidemic [5]. Pillar one of the strategy – ‘integrated patient centred care and prevention’, requires countries to improve the diagnosis and management of TB in high-risk groups [5]. It is imperative that TB control strategies are adapted to fit the local epidemiology to ensure effective and efficient use of limited resources and ensure vulnerable and high-risk groups within the population are reached and that their health needs are adequately addressed. While it is clear that the burden of TB in Sabah is disproportionately high, there is little published data characterising the local TB epidemic. The most recent report presents data from two decades ago [8]. The aim of this study is to describe the epidemiology of TB in Sabah to better identify risk groups and hotspots of TB transmission.

## Methods

### Data sources

In Sabah, all microbiologically confirmed and clinical cases of TB must be notified to the State Health Department where they are recorded in a secure electronic database (myTB). Case-based data in myTB included demographic, clinical, diagnostic and treatment outcome data. The myTB database records information on three specific TB risk factors – HIV co-infection, diabetes and smoking. Population data used to calculate incidence rates was obtained from the Malaysian Department of Statistics and data on national TB notifications in Malaysia was obtained from the World Health Organization (WHO) [11].

### Definitions

All notified cases of TB disease in Sabah State, Malaysia, with a date of diagnosis between 1 January 2012 and 31 December 2018, recorded in the myTB database, were included in the study. The study period was based on the availability of complete calendar years of data at the time of the study. Bacteriologically confirmed TB was defined as a case in whom a biological specimen is positive by smear microscopy, culture or WHO recommended rapid diagnostic test such as GeneXpert®. Clinically-diagnosed TB includes cases who do not meet the criteria for bacteriologically confirmed cases, but in whom a clinician has diagnosed TB and decided to provide a full course of anti-TB treatment [12]. Radiological severity was assessed using established grading criteria and classified as no lesion, minimal lesion, moderately advanced or far advanced [13, 14].

Treatment outcome was classified based on WHO reporting guidelines [12], as treatment success, failure, died, lost to follow-up or not evaluated. ‘Treatment success’ included cases confirmed cured by negative culture at completion of treatment, or who successfully completed the full course of treatment. ‘Not evaluated’ included patients who were transferred out or where the treatment outcome was not recorded. Treatment success was calculated as the proportion of all registered cases who completed treatment or were cured. For cases of multi-drug resistant TB (MDR-TB), treatment outcome was assessed for years 2012–2016 to account for completion of longer treatment regimens, and incomplete outcome data for 2017 and 2018 at the time of analysis.

### Data analysis

We determined numbers of cases of drug-sensitive and multi-drug resistant TB and notification rate of drug-sensitive disease per 100 000 population, by year and district of residence. Using national notification data, we calculated the proportion of all cases in Malaysia occurring in Sabah State. Cases were characterised by age, sex, country of birth and Malaysian citizenship status. We assessed the prevalence of the three reported risk factors among TB cases. Clinical and diagnostic information was used to determine the proportion of cases diagnosed clinically or with bacteriological confirmation, the proportion with pulmonary versus extrapulmonary disease, and various sites of infection. Site of infection data was available for years 2012 to 2017 only.

We assessed the proportion of new versus previously treated cases, the method of case detection and type of diagnosing heath facility, the proportion of pulmonary sputum smear positive cases, and the proportion with moderate to advanced disease on chest X-ray. We assessed treatment outcomes, including sputum smear conversion during treatment and final treatment outcome. The Chi-squared test was used for determining the difference between two proportions.

Descriptive analysis of demographic, epidemiological and clinical data was undertaken using R statistical software (version 3.6.1, R Core Team, Vienna, Austria, available at https://www.r-project.org/). Maps were produced using QGIS (version 3.8.2, QGIS Development Team, Open Source Geospatial Foundation Project, available at https://qgis.org/en/site/).

## Results

**Fig. 1:**
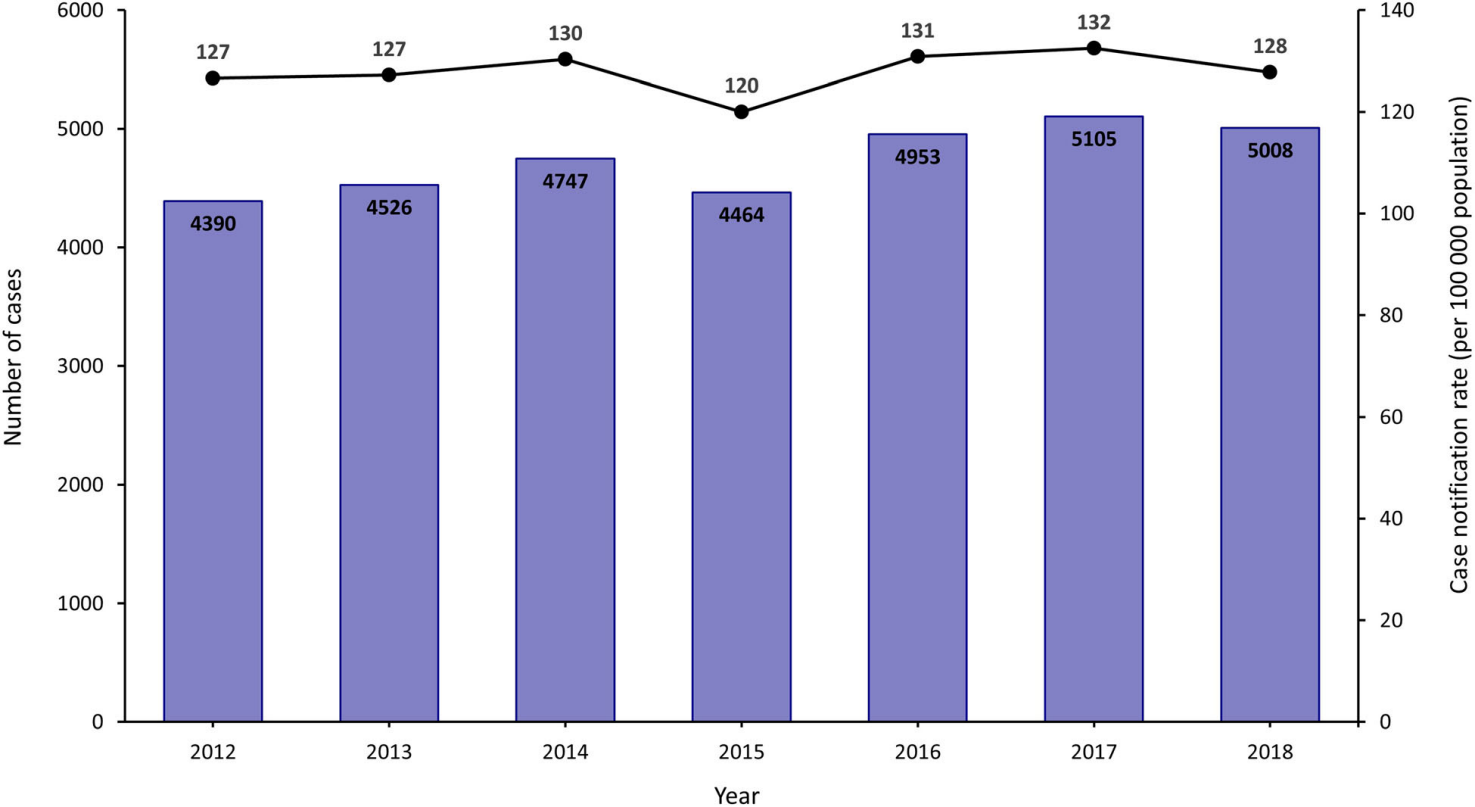
Number of notified tuberculosis cases and case notification rate by year, Sabah, 2012–2018

Between 2012 and 2018, there were 33 193 TB cases reported to the Sabah State TB surveillance database (myTB). This equated to an overall notification rate of 128 cases (range: 120–133) per 100 000 population (Fig. 1), and 19.7% (range: 18.9–20.1%) of the total reported TB burden nationally in Malaysia.

**Fig. 2:**
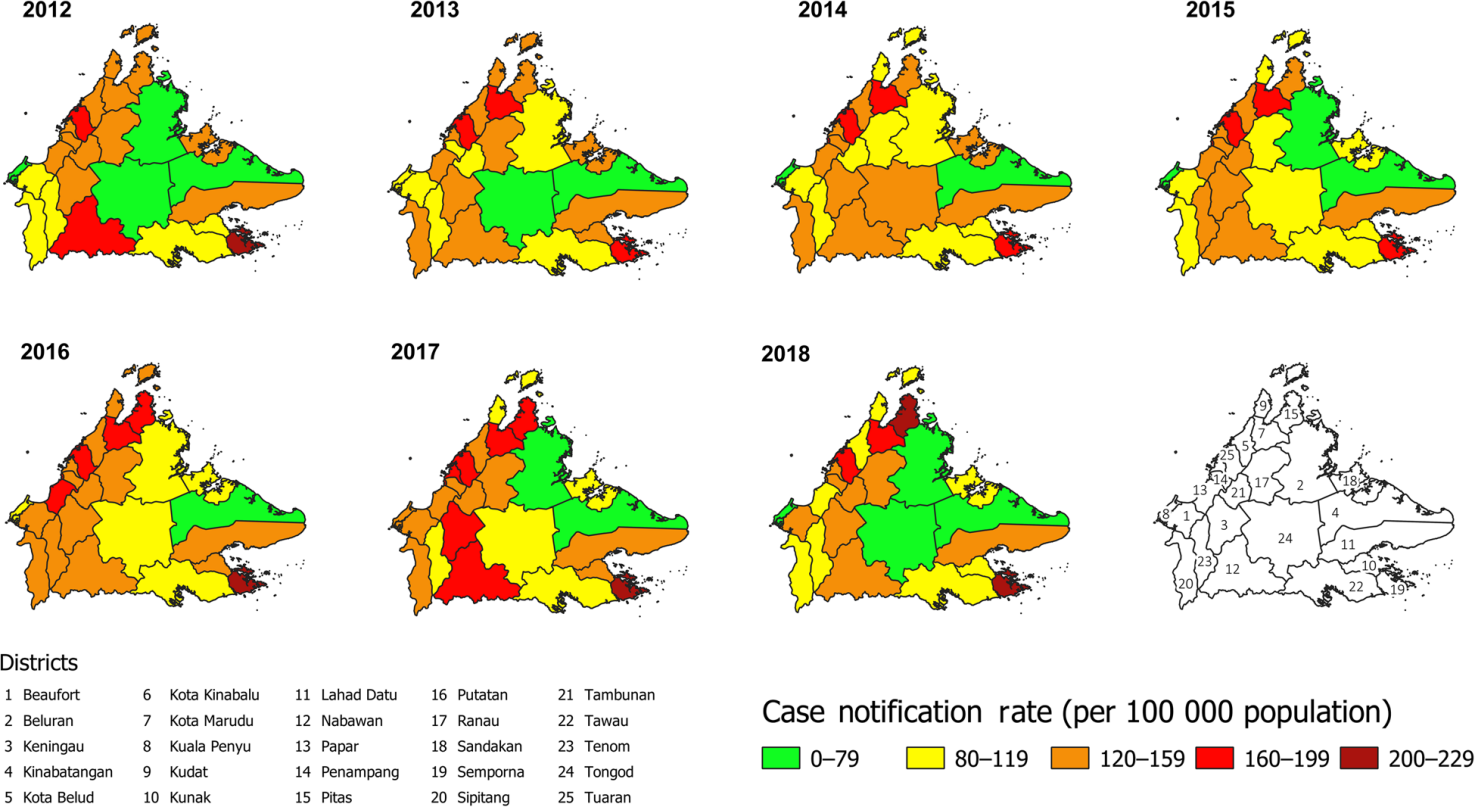
Tuberculosis case notification rate by district and year, Sabah, 2012–2018

TB case numbers and notification rates varied substantially between the 25 districts of Sabah. Kota Kinabalu, the state capital and largest population centre of Sabah, had the highest annual number of TB cases in each study year (*n* = 904 in 2018), accounting for 15–18% of reported cases. The highest case notification rates were seen in the Eastern coastal district of Semporna and northern coast district of Pitas, both with 228 cases per 100 000 population in 2018. The lowest reported rates in 2018 were in the central eastern district of Kinabatangan (56 cases per 100 000) and interior district of Tongod (53 cases per 100 000), both of which are characterised by large areas of jungle and remote rural communities (Fig. 2).

### Demographic characteristics

The median age of TB cases was 38 years. Case notification rates increased steadily with age from a low of 23 cases per 100 000 among children aged 5–14 years, to a high of 402 cases per 100 000 among adults aged 65 years and older. Only 1.6% of cases were among children aged less than 5 years, and 4.6% of cases were children less than 15 years. Further, the proportion of total cases that were children decreased from 2012 to 2018: by 2018, only 1.2% of cases were < 5 years and 3.6% were < 15 years.

**Fig. 3:**
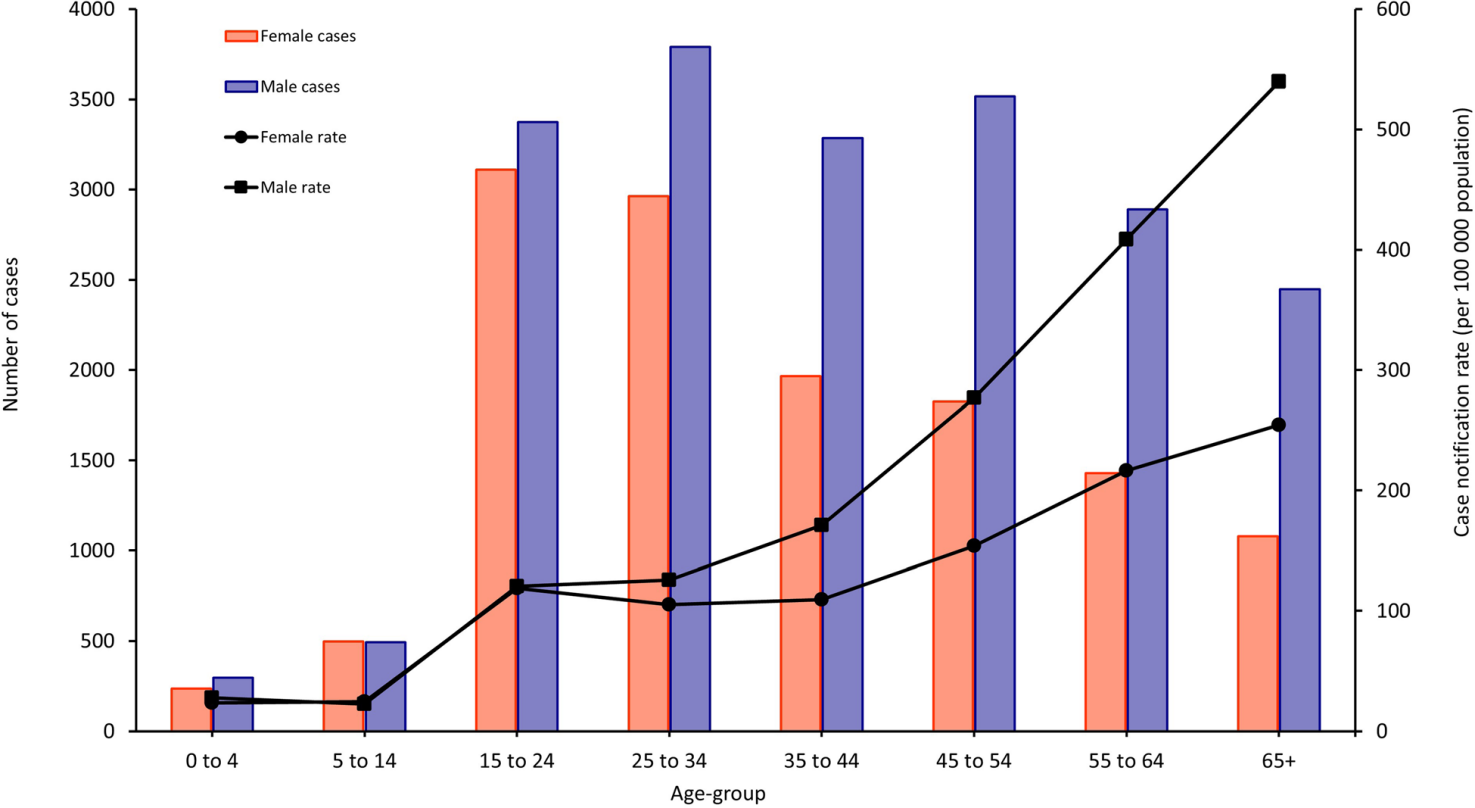
Number of notified tuberculosis cases and case notification rate by age-group and sex, Sabah, 2012–2018

There was substantial gender disparity among reported TB cases; 60% of cases were male. This was most notable in older age groups – in particular, those aged ≥ 55 years had a male to female notification ratio of 2.1:1. In both males and females, notification rates increased with age, but from adults aged 25 years and older, there was increasing disparity between sexes. The highest notification rates were seen in males aged 65 years and older at 540 cases per 100 000 population (compared to 254 cases per 100 000 in females in the same age group). There was little difference in the number of notifications between males and females among children less than 15 years (Fig. 3).

**Fig. 4:**
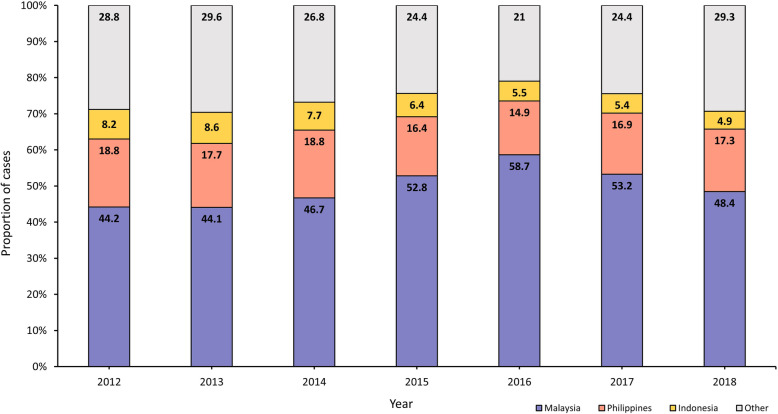
Proportion of notified tuberculosis cases by country of birth, Sabah, 2012–2018

**Fig. 5:**
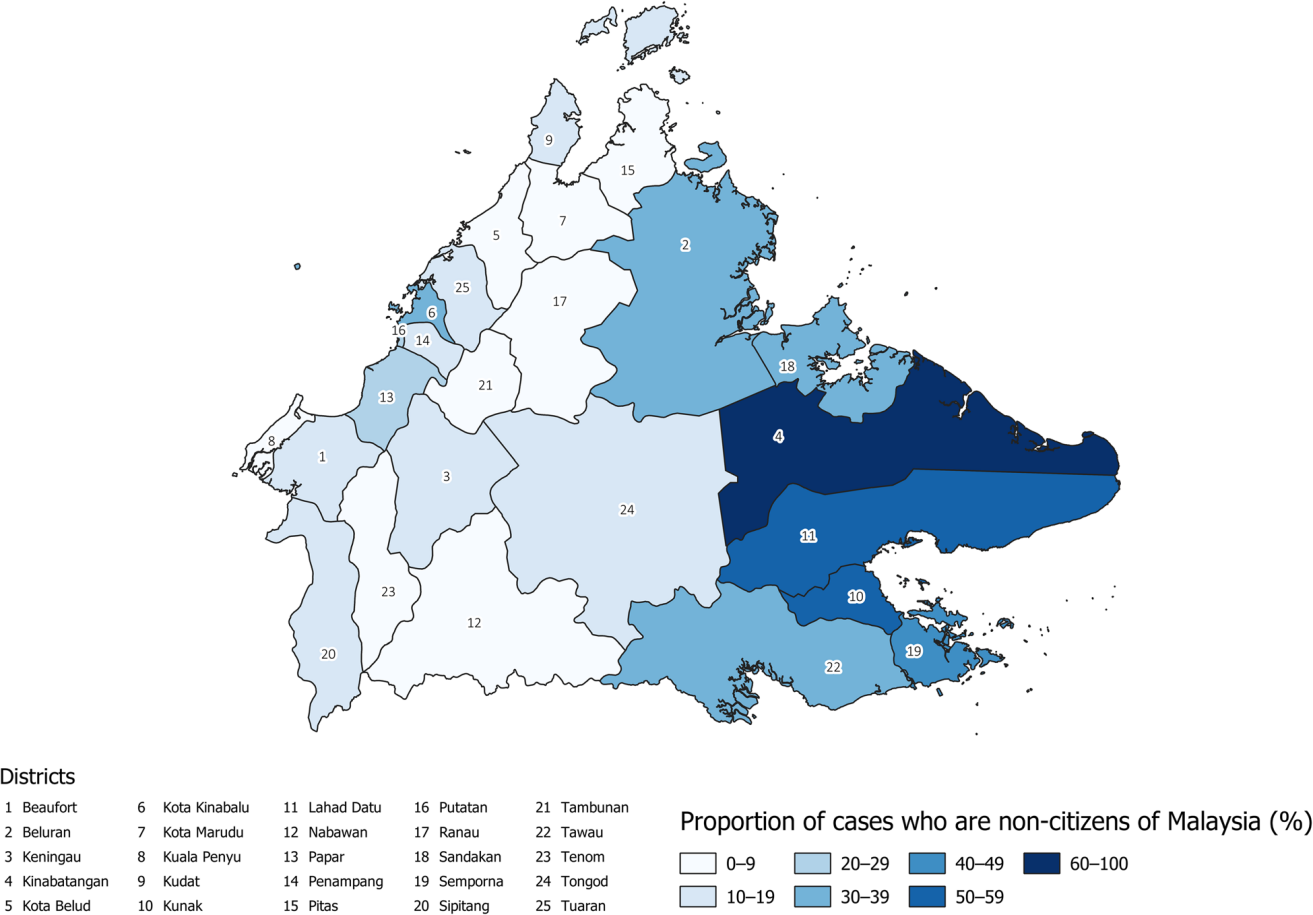
Proportion of notified tuberculosis cases who are non-citizens of Malaysia, by district, Sabah, 2012–2018

Over the course of the study period, 50% of cases were born in Malaysia. Of cases born outside Malaysia, the majority were born in the Philippines (17%) and Indonesia (7%). The remaining 26% represented a range of other nationalities. There was some variation in the distribution year to year, though no obvious trend (Fig. 4). Most cases (71%) were Malaysian citizens, and no trend in the proportion of cases with or without Malaysian citizenship was observed over the study period. However, there was a substantial difference by district of residence in the proportion of cases who were non-citizens – ranging from < 5% of cases in Nabawan, Kota Belud and Pitas, to > 50% of cases in the eastern districts of Lahad Datu and Kunak and 62% of cases in Kinabatangan (Fig. 5). Among overseas-born cases, only 14% had been in Malaysia < 5 years at their time of diagnosis; 52% of overseas-born cases had a length of stay in Malaysia of 10–29 years at their time of diagnosis.

Over the study period, monthly median household income of TB cases was 1000 Malaysian Ringgit (MYR) (equivalent to ~USD 250).

### Case detection and diagnosis

**Fig. 6:**
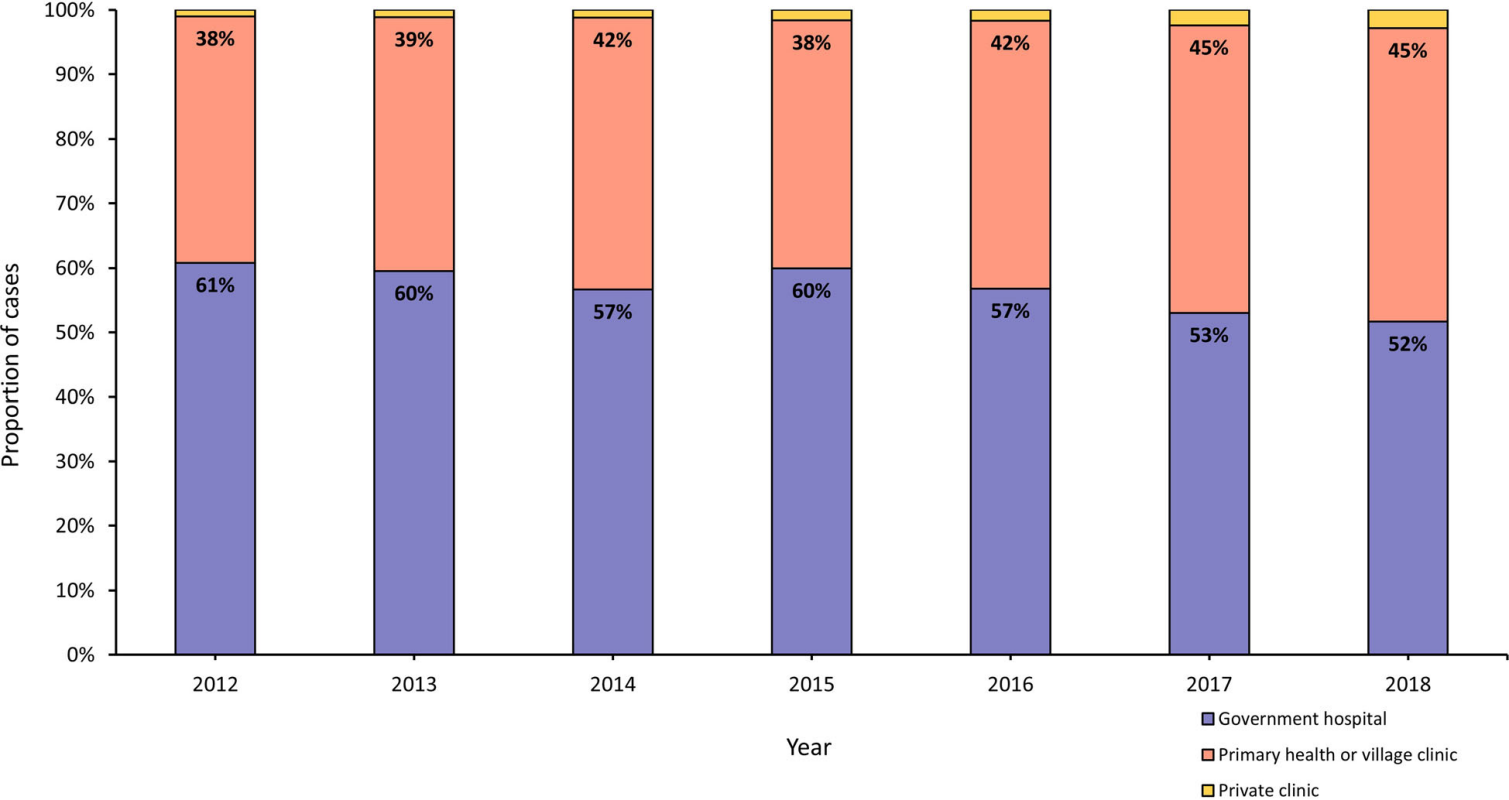
Proportion of notified tuberculosis cases by diagnosing facility type, Sabah, 2012–2018

Overall, 57% of TB cases were diagnosed in government hospitals, with 42% diagnosed in public primary health or village clinics and 2% diagnosed in private facilities. Over time, there was a decrease in the proportion of cases diagnosed in government hospitals, from 61% of cases in 2012, down to 52% in 2018. A corresponding increase in diagnoses occurred in primary care (primary health and village clinics) and a small increase at private facilities (clinics and hospitals) (Fig. 6).

Between 2012 and 2018, most cases (89%) were detected passively. Of those that were diagnosed through active case finding, 74% (*n* = 2244) were detected during contact investigation – equivalent to 6.8% of all TB cases during this period. The remaining 26% of cases were detected through various screening mechanisms, such as community or occupational screening – equivalent to 4.2% of all TB cases during this period. The overall proportion of TB cases detected through community screening did increase from 2014 onwards, though accounted for only a very small proportion of overall case detection (reaching just 1.7% in 2018).

Seventy-three percent of TB cases between 2012 and 2018 were bacteriologically confirmed (range: 70–74%). However, among children less than 5 years, only 19% were bacteriologically confirmed. Among all cases during this period, only 74% were tested by culture, varying year to year from 51 to 87%. Culture positivity was low at 24% overall, ranging from 11 to 29% between years. Culture positivity among pulmonary cases only was 26%. A total of 403 cases were tested by GeneXpert® during the study period, equating to test coverage of only 1.2% (range: 0.3–3.0%). GeneXpert® positivity was higher than culture at 66% overall, but also varied substantially from 0% (of 12 samples tested) in 2012 to 89% (of 149 samples tested) in 2016. Most cases (97%) had smear microscopy results available, and this was consistent across the period (Table 1).

**Table 1:** Diagnostic results of notified TB cases by year, Sabah, 2012–2018

Year	Bacteriologically confirmed	GeneXpert®			Culture			Smear microscopy		
		Number of cases tested	Proportion of cases tested	Proportion of tests positive	Number of cases tested	Proportion of cases tested	Proportion of tests positive	Number of cases tested	Proportion of cases tested	Proportion of tests positive
2012	74.3%	12	0.3%	0.0%	2232	50.8%	11.3%	4227	96.3%	76.6%
2013	73.1%	15	0.3%	6.7%	3223	71.2%	13.5%	4363	96.4%	75.1%
2014	72.8%	27	0.6%	29.6%	4108	86.5%	22.4%	4598	96.9%	74.5%
2015	74.0%	80	1.8%	41.3%	3620	81.1%	28.4%	4328	97.0%	75.3%
2016	74.1%	149	3.0%	88.6%	3971	80.2%	29.2%	4827	97.5%	74.0%
2017	70.0%	74	1.4%	71.6%	3981	78.0%	28.6%	5016	98.3%	69.8%
2018	70.0%	46	0.9%	82.6%	3375	67.4%	26.1%	4904	97.9%	70.5%
Total	72.6%	403	1.2%	65.8%	24 510	73.8%	23.7%	32 263	97.2%	73.5%

Among pulmonary cases, sputum smear positivity at diagnosis was high at 81% between 2012 and 2018, however there was a decreasing trend in sputum smear positivity from 84% in 2012 to 79% in 2018. When broken down by citizenship status, 85% of non-citizens with pulmonary TB were sputum smear positive at diagnosis, significantly higher than the proportion of Malaysian citizens (79%) who were smear positive at diagnosis (*χ*^*2*^ = 145.9, *P* < 0.0001).

Chest X-ray coverage of pulmonary cases was high across the study period, with 97% of cases having a chest X-ray finding at diagnosis recorded. Fifty-one percent of pulmonary cases had moderately advanced (44%) or far advanced (6%) disease at diagnosis, with the remaining 49% having minimal lesions or no lesion present on chest X-ray. Malaysian citizens were significantly less likely to have moderate or advanced disease (46%) on chest X-ray at diagnosis compared with non-citizens (57%) (*χ*^*2*^ = 362.7, *P* < 0.0001).

### Case classification

Of the 33 193 cases notified between 2012 and 2018, 94% were new TB cases and 6% were reported as previously treated (range: 5.4–7.2%). Eighty-nine percent of cases had pulmonary disease, with the remaining 11% having extrapulmonary disease only. There was no change over time in the proportion of extrapulmonary TB. Among 4166 cases of TB with extrapulmonary involvement reported between 2012 and 2017, the most common sites of infection were the lymphatic system (33%), pleura (17%), bone, joints and soft tissue (15%), and gastrointestinal system (13%). Ten percent of cases had central nervous system disease, including TB meningitis, and 7% with extrapulmonary involvement had disseminated disease. The genitourinary system, pericardium and eyes accounted for very small numbers (< 2%).

### Risk factors

Overall, 7% of TB cases were recorded as having diabetes, increasing from 5% of cases in 2012 to 8% in 2016–2018. There was no difference in diabetes prevalence by gender. Just under one-third of TB cases (31%) were recorded as smokers, the prevalence of which remained unchanged. The proportion of cases who smoked varied greatly by gender, with 48% of male TB cases recorded as smokers, versus just 4% of female cases recorded as smokers. Among those aged 35 years and older, the male to female ratio decreased from 2.0 to 1.1 when smokers were excluded. (Supplementary Figure 1) An average of 48 cases of TB per year (approximately 1% of cases) occurred in healthcare workers. HIV testing among TB cases was high, with results of HIV testing recorded for more than 99% of TB cases. HIV prevalence among TB cases was low, with just 1.8% of cases between 2012 and 2018 recorded as having HIV co-infection (range: 1.6–2.0%). Among TB cases with HIV co-infection, 43% received antiretroviral therapy (ART) (range: 29–63%).

### Drug resistance

A total of 87 cases of MDR-TB were reported in Sabah between 2012 and 2018 (range: 9–16 cases per year), equivalent to 0.3% of all cases. MDR-TB prevalence was 0.1% (*n* = 42) among new cases and 2.2% (*n* = 45) among previously treated cases. One-third of MDR-TB cases occurred in just two districts, Kota Kinabalu (17%, *n* = 15) and Tawau (16%, *n* = 14).

### Treatment outcomes

Treatment success among cases of drug-sensitive TB was 83% (range: 81–85%) for the period of 2012 to 2018. Less than 1% of cases failed treatment and 8% of cases died. Among the remainder, 2% of cases were recorded as lost to follow-up and 6% of cases were not evaluated. There was no substantial change in treatment outcome among drug-sensitive cases over the study period. Among cases of drug-sensitive TB, 84% of new cases successfully completed treatment, versus 77% of previously treated patients. Among cases of MDR-TB, treatment success was much lower, with only 36% (range: 25–45%) of cases reported between 2012 and 2016 cured or successfully completing treatment. Three percent of MDR-TB cases died, 31% failed treatment, and 7% were lost to follow-up. The remainder were not evaluated for treatment outcome. Given the small number of MDR-TB cases, it was not possible to determine any trend in treatment outcomes over time.

## Discussion

Our study provides a detailed sub-national analysis of the epidemiological situation of TB in Sabah, Malaysia in recent years. Findings highlight the high burden of disease in Sabah compared with the rest of Malaysia. In 2018, the case notification rate in Sabah was 47% higher than that of the national case notification rate of Malaysia [11, 15]. We note that case notification rates do not represent the true incidence of TB. In Malaysia, 13% of estimated incident TB cases in 2018 were not notified [11]. Sub-national estimates of TB incidence in Sabah are not available, however we would anticipate that there is a substantial gap between the incidence and notification rates within Sabah, at least equivalent to that seen at the national level. As such, the case notification rates presented are likely to underestimate the true burden of disease.

Sabah State is a setting which poses unique challenges to Malaysia’s national TB control program. Sabah is less economically developed than peninsular Malaysia, has an interior which can be topographically challenging and difficult to access for healthcare provision, has substantial communities of marginalised persons living in overcrowded informal settlements, and a porous land/sea border with the Philippines [16]. Our results show that while Sabah has a moderate overall burden of TB, there were geographic hotspots of disease – particularly the districts of: Semporna, Pitas, Kota Marudu, Tuaran, and Kota Kinabalu. These districts correspond with areas of frequent cross-border movement between Malaysia and the Philippines (Semporna and Pitas) and the more populous urbanised districts of north-western Sabah (Tuaran, Kota Kinabalu and Kota Marudu).

Given the high burden of TB in Sabah’s nearest two neighbours, the Philippines and Indonesia (incidences of 554 and 316 cases per 100 000 population respectively) [11], and the frequent movement of people across these international borders, there is an evident need for the Malaysian healthcare system to actively engage international arrivals in no-cost TB prevention and early detection strategies. It is difficult however, to ascertain the relative burdens of imported versus locally acquired TB disease. Our finding of geographic areas with a high proportion of cases among non-citizens, and high-burden urban areas with a relatively low proportion of cases among non-citizens, may support a hypothesis of seeding of overseas acquired disease in Sabah, with amplification of transmission occurring in densely populated urban centres. Investment in healthcare for non-citizens could therefore have a major beneficial impact on overall TB rates. Previous studies have shown that the majority of TB cases occur within 2 years of infection [17, 18]. However, we found that among overseas born TB cases, the majority developed TB five or more years after arrival in Sabah, further supporting the likelihood that transmission of TB, even among overseas born cases, is occurring locally.

Sabah’s migrant population, especially those who are refugees, from stateless minority groups and illegal or undocumented migrants face substantial challenges to accessing healthcare and social protection – including financial, legal, language and physical access barriers [19–21]. The finding that non-citizens were significantly more likely to have advanced disease on chest X-ray and be sputum smear positive at presentation, supports the hypothesis that these groups face additional barriers to receiving timely diagnosis and treatment. We found that monthly household income of TB cases in Sabah (MYR 1000 or USD 250) was around one-quarter the median monthly household income across Sabah State (MYR 4110 or USD 1031 in 2016) [22]. This highlights the risk of financial barriers limiting access to diagnosis and care.

The United Nations Sustainable Development Goals encourage countries to achieve universal health coverage and WHO’s End TB Strategy reinforces the role of universal health coverage in ensuring equal and unhindered access to early diagnosis and treatment of TB [5, 23]. Understanding the barriers that both non-citizens and citizens of Malaysia may face in accessing TB services is essential, followed by engagement of communities and reorientation of services to ensure patient-centred care [5]. We note that over the study period there has been a steady shift in diagnosis of TB from government hospitals to primary health and village clinics and the private sector. This shift may indicate a decentralisation of services which could improve access to TB diagnosis and care. It is however important that the TB program engages with and equips primary health, village clinics and private facilities with the support they need to increase their role in TB care.

Our results highlight key gaps in the detection of TB in Sabah. We found that case detection among children was very low, with children aged less than 15 years accounting for only 4.6% of cases, despite that age-group accounting for 24% of the Sabah population and global estimates of disease burden suggesting that this age-group accounts for 11% of all TB cases [1]. The decreasing trend in proportion of TB cases who are children is concerning. Further investigation is needed to determine the reasons for this. As our findings show, the majority of cases among children are diagnosed clinically. This highlights the importance of clinicians being trained in symptom-based screening and diagnosis of TB in children and collection of appropriate diagnostic samples such as gastric lavage and induced sputum. Given the likely under-diagnosis of childhood TB in Sabah, TB contact investigation should be strengthened to improve early detection of childhood TB, while enabling scale-up of treatment for TB infection to prevent future cases among high-risk contacts [24].

Uptake of WHO-recommended rapid diagnostics (GeneXpert®) for diagnosis of TB was limited. However, at the time of the study, national guidelines did not recommend GeneXpert® as a first-line diagnostic test for all suspected drug-sensitive TB cases. Both culture and smear microscopy were mandatory components of the diagnostic algorithm in Malaysia [25, 26]. Rapidly increasing GeneXpert® coverage should be a priority, especially given that a quarter of cases were not tested by culture, and of those cases that were, culture positivity was extremely low (26% among pulmonary cases). While culture negative pulmonary TB can be an indicator of early disease [27], the concurrent findings of high sputum smear positivity and high proportion of cases with moderate-advanced disease on chest X-ray, suggest the low culture positivity rate may be related to sub-optimal test sensitivity in this setting, noted in Kota Kinabalu previously [28].

The low coverage of GeneXpert® and high proportion of culture negative or not tested cases, raises concern of drug-resistant TB being missed. The 2018 WHO estimates for MDR-TB in Malaysia for both new (1.5%) and previously treated cases (3.1%) [11], are considerably higher than what we observed in Sabah. Fast tracking the scale up GeneXpert® coverage would not only aid in improving confirmation of drug-sensitive TB, but help ensure that rifampicin resistance is detected. While current cases of MDR-TB are low, the lack of testing limits our confidence in whether all cases are being detected. Adding to this concern are the poor treatment outcomes recorded among MDR-TB cases. Given one-third of all MDR-TB cases stopped or failed treatment, there is a risk that these cases may continue to be infectious and contribute to primary transmission of drug resistance in Sabah. Efforts to improve treatment outcomes among MDR-TB cases, such as through improved treatment supervision and monitoring, consideration of new all oral or standardised shorter regimens, and patient support measures should be prioritised [29].

Understanding the local epidemiology in this unique context is critical to implementing effective measures for TB control and prevention. TB notification data is the best available source of data in Sabah for describing the local epidemiological situation. However, the findings are potentially limited by the quality and completeness of data recording and entry. Routine surveillance data is inherently limited in the breadth of information that can be collected. We note that collection of additional data, such as other known risk factors for TB would further enhance our understanding of the epidemiology. Malaysia has a complex system of both paper based and electronic mechanisms for collection of patient data and notification of TB, and enumeration of cases may be imperfect. There was no scope to cross-check data from the myTB database against primary records. However, the State Department of Health undertakes regular auditing and cleaning of data to improve its quality and completeness. Thus, our findings are likely to also underestimate the true burden of disease, particularly in some groups of the population which face additional barriers to care, such as those living in remote areas and migrants, particularly those without legal status.

## Conclusions

This study highlights the value of detailed sub-national review of TB notification data to evaluate programmatic outcomes. The TB burden remains disproportionately high in Sabah, compared with peninsular Malaysia. Within Sabah, geographic hotspots of TB correspond with migration hubs and dense urbanised population centres. Further micro-level analysis of the epidemiology and service utilisation within these centres may assist in formulating strategies for targeted active case finding activity. However, given the evidence of late diagnosis of TB, particularly among non-citizens, priority should be given to ensuring universal health coverage and removal of any social, financial, legal, linguistic or other barriers to care. There is an urgent need to scale up use of GeneXpert® in Sabah. This would help improve the diagnostic gap in children and ensure that rifampicin-resistance is detected.

## Supplementary information

**Additional file 1.** Supplementary Figure 1. Number of notified TB cases and case notification rate among non-smokers by age-group and sex, Sabah, 2012–2018.

## Data Availability

The data that support the findings of this study are available from the Sabah State Department of Health, but restrictions apply to the availability of these data, which were used under license for the current study, and so are not publicly available. Data may however be available from the authors upon reasonable request and with permission of the Sabah State Department of Health.

## Abbreviations

HIV: Human immunodeficiency virus
MDR-TB: Multi-drug resistant tuberculosis
MYR: Malaysian Ringgit
TB: Tuberculosis
USD: United States Dollar
WHO: World Health Organization

## References

[1] World Health Organization (2019). Global Tuberculosis Report 2019.

[2] The World Bank (2019). World Bank Country and Lending Groups.

[3] Fisher D, Wilder-Smith A (2020). The global community needs to swiftly ramp up the response to contain COVID-19. Lancet..

[4] Iyawoo K (2004). Tuberculosis in Malaysia: problems and prospect of treatment and control. Tuberculosis..

[5] World Health Organization (2018). The end TB strategy: global strategy and targets for tuberculosis prevention, care and control after 2015.

[6] Rundi C, Fielding K, Godfrey-Faussett P, Rodrigues LC, Mangtani P (2011). Delays in seeking treatment for symptomatic tuberculosis in Sabah, East Malaysia: factors for patient delay. Int J Tuberc Lung Dis.

[7] Paul DC, Chew A, Yau NL, Hollip KC, Tangkanggau F, Lasimin S, et al. Multidrug resistant *Mycobacterium tuberculosis* complex: a laboratory perspective (conference poster abstract). 7th Public Health Colloquium, Sabah State Health Department; Ming Garden Hotel, Kota Kinabalu, Malaysia, 21–22 November 2012.

[8] Dony JF, Ahmad J, Khen Tiong Y (2004). Epidemiology of tuberculosis and leprosy, Sabah, Malaysia. Tuberculosis.

[9] Rashid Ali MR, Parameswaran U, William T, Bird E, Wilkes CS, Lee WK (2015). A prospective study of tuberculosis drug susceptibility in Sabah, Malaysia, and an algorithm for management of isoniazid resistance. J Trop Med.

[10] Muhammad Redzwan SR, Ralph AP, Sivaraman Kannan KK, William T (2015). Individualised second line anti-tuberculous therapy for an extensively resistant pulmonary tuberculosis (XDR PTB) in East Malaysia. Med J Malaysia.

[11] World Health Organization (2020). Tuberculosis country profiles.

[12] World Health Organization (2013). Definitions and reporting framework for tuberculosis – 2013 revision.

[13] Seaton A, Seaton D, Leitch AG (1989). Diagnostic imaging. Crofton and Douglas’s respiratory diseases.

[14] Deniz O, Tozkoparan E, Yonem A, Ciftci F, Bozkanat E, Cakir E (2005). Low parathormone levels and hypercalcaemia in patients with pulmonary tuberculosis: relation to radiological extent of disease and tuberculin skin test. Int J Tuberc Lung Dis.

[15] World Health Organization (2020). Global tuberculosis database.

[16] Dollah R, Wan Hassan WS, Peters D, Othman Z (2016). Old threats, new approach and national security in Malaysia: issues and challenges in dealing with cross-border crime in east coast of Sabah. Mediterr J Soc Sci.

[17] Behr MA, Edelstein PH, Ramakrishnan L (2018). Revisiting the timetable of tuberculosis. BMJ..

[18] Borgdorff MW, Sebek M, Geskus RB, Kremer K, Kalisvaart N, van Soolingen D (2011). The incubation period distribution of tuberculosis estimated with a molecular epidemiological approach. J Epidemiol.

[19] Loganathan T, Rui D, Ng C-W, Pocock NS (2019). Breaking down the barriers: understanding migrant workers’ access to healthcare in Malaysia. PLoS One.

[20] Lasimbang H, Tong WT, Low WY (2015). Migrant workers in Sabah, East Malaysia: the importance of legislation and policy to upheld equity on sexual and reproductive health and rights. Best Pract Res Clin Obstet Gynaecol.

[21] Kheng KS, Teo J, Yong Kek PY, Swee Fong TS, Quah H, Bawi J (2020). Proposing a non-citizens health act for Malaysia.

[22] Department of Statistics Malaysia (2017). Report of household income and basic amenities survey 2016.

[23] United Nations (2020). Sustainable development goals knowledge platform.

[24] Dodd PJ, Yuen CM, Becerra MC, Revill P, Jenkins HE, Seddon JA (2018). Potential effect of household contact management on childhood tuberculosis: a mathematical modelling study. Lancet Glob Health.

[25] Ministry of Health Malaysia (2012). Management of tuberculosis.

[26] Ministry of Health Malaysia (2016). Clinical practice guidelines: management of drug resistant tuberculosis.

[27] Achkar JM, Jenny-Avital ER (2011). Incipient and subclinical tuberculosis: defining early disease states in the context of host immune response. J Infect Dis.

[28] Lowbridge C, Fadhil SAM, Krishnan GD, Schimann E, Karuppan RM, Sriram N (2020). How can gastro-intestinal tuberculosis diagnosis be improved? A prospective cohort study. BMC Infect Dis.

[29] World Health Organization (2019). WHO consolidated guidelines on drug-resistant tuberculosis treatment.

