# Supplementary figures and images for: Epidemiology of tuberculosis in Sabah, Malaysia, 2012–2018
Source: Infect Dis Poverty. 2020 Aug 26;9:119. doi: 10.1186/s40249-020-00739-7 (PMC7447595; doi:10.1186/s40249-020-00739-7)

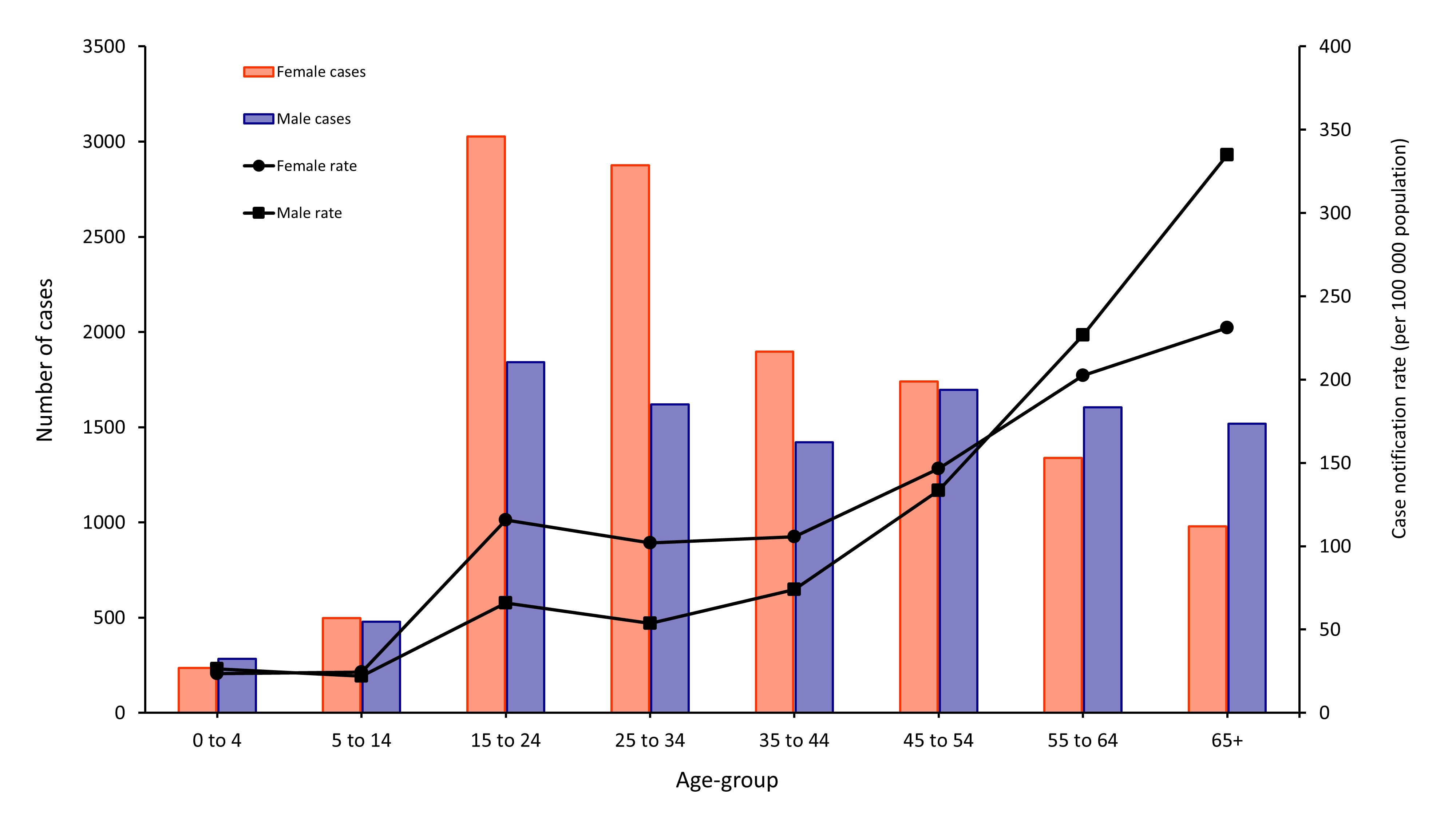

Supplement: Supplementary file 1 — Additional file 1. Supplementary Figure 1. Number of notified TB cases and case notification rate among non-smokers by age-group and sex, Sabah, 2012–2018. [file 40249_2020_739_MOESM1_ESM.jpg]
